# Primary Metabolite Chromatographic Profiling as a Tool for Chemotaxonomic Classification of Seeds from Berry Fruits

**DOI:** 10.17113/ftb.60.03.22.7505

**Published:** 2022-09

**Authors:** Đurđa Krstić, Tomislav Tosti, Saša Đurović, Milica Fotirić Akšić, Boban Đorđević, Dušanka Milojković-Opsenica, Filip Andrić, Jelena Trifković

**Affiliations:** 1 University of Belgrade, Faculty of Chemistry, Studentski trg 12-16, 11158 Belgrade, Serbia; 2 Institute of General and Physical Chemistry, Studentski trg 12/V, 11158 Belgrade, Serbia; 3 University of Belgrade, Faculty of Agriculture, Nemanjina 6, 11080 Belgrade, Serbia

**Keywords:** sugar, lipid and fatty acid identification, chemical fingerprint, berry seeds, chromatography techniques

## Abstract

**Research background:**

Considering the importance of consumption of berry fruits with proven health-beneficial properties and difficulties in quality control of products of specific botanical and geographic origin, a fingerprint method was developed, based on advanced data analysis (pattern recognition, classification), in order to relate the variability of nutrients in the selected cultivars to primary metabolite profile.

**Experimental approach:**

Forty-five samples of genuine berry fruit cultivars (strawberry, raspberry, blackberry, black currant, blueberry, gooseberry, chokeberry, cape gooseberry and goji berry) were characterized according to chromatographic profiles of primary metabolites (sugars, lipids and fatty acids) obtained by three chromatographic techniques (high-performance thin-layer chromatography, gas chromatography coupled to mass spectrometry, and high-performance anion-exchange chromatography with pulsed amperometric detection).

**Results and conclusions:**

Comprehensive analysis allowed monitoring and identification of metabolites belonging to polar lipids, mono-, di- and triacylglycerols, free fatty acids, free sterols, sterol esters, mono- to heptasaccharides and sugar alcohols. Chemical fingerprint of berry seeds showed the uniformity of primary metabolites within each fruit species, but revealed differences depending on the botanical origin. All three chromatographic methods provided a discriminative, informative and predictive metabolomics methodology, which proved to be useful for chemotaxonomic classification.

**Novelty and scientific contribution:**

A novel methodology for the identification of bioactive compounds from primary metabolites of natural products was described. The proposed untargeted metabolite profiling approach could be used in the future as a routine method for tracing of novel bioactive compounds. The knowledge of metabolite composition obtained in this study can provide a better assessment of genotypic and phenotypic differences between berry fruit species and varieties, and could contribute to the development of new breeding programs.

## INTRODUCTION

Searching for the specific properties as a characteristic of certain taxon is a main task of authenticity assessment. The chemical structure of the metabolites and their biosynthetic pathways are specific and restricted to taxonomically related species and therefore useful for their characterization and classification (*1*). Chemotaxonomy, as an approach to plant classification based on their chemical constituents, is nowadays widely used.

Secondary metabolites are widely used for chemotaxonomic classification due to the high variation in chemical diversity, distribution and function (*1*). Glycosides, alkaloids, volatile oils, flavonoids, plant phenols and terpenoids are proven to be good markers of origin because of their specific functions and restricted occurrence in the plants. Primary metabolites, however, are involved in fundamental metabolic processes such as photosynthesis, nutrient assimilation and respiration, and they have essential role in plant growth, development and stress adaptation (*2*). They are ubiquitous, rarely specific, and therefore mainly inappropriate for metabolomic studies (*3*).

Contrary to traditional taxonomical methods based on morphological and anatomical features, chemotaxonomy is characterized by easier working methodology and higher sensitivity due to the advanced analytical techniques for detection of even trace amount of compounds. Separation techniques such as high-performance (HP) or ultra-performance (UP) liquid chromatography (LC), gas chromatography (GC), capillary electrophoresis (CE), coupled with detection techniques such as mass spectrometry (MS), nuclear magnetic resonance (NMR) and near infrared (NIR) spectrometry are widely used for both metabolomic approaches, targeted and untargeted metabolite profiling (*4*-*6*). Due to the complex nature of plants, a chemical fingerprint that establishes a characteristic chemical pattern of an extract is sometimes better approach for authenticity assessment than targeted analysis, which is restricted to quantitative analysis of a class of compounds that are related to a specific pathway (*7*). However, although metabolite profiling involves rapid analysis, great amount of data obtained from the profiles requires the knowledge in advanced data analysis, signal processing and statistics to efficiently extract the maximum useful information. Multivariate statistical techniques developed for analytical chemistry have been widely used in food analysis for authenticity, functionality and bioactivity assessments, and food safety (*8*).

A unique phytochemical composition of berry seed has been gaining attention in recent years as a potential source of functional ingredients in food and pharmaceutical industry (*9*, *10*). The composition of the seed oil differs among the various berry species, while it tends to be characteristic within the same genus or family (*11*, *12*). Sugar content varies depending on ripeness, agroecological and cultivation conditions, fertilization, temperature and genotype (*13*). They are extraordinary sources of sugars, acids, polyphenols, carotenoids, tannins, vitamins (A, B1, B2, C, E, and B3), dietary fibre and other phytochemicals which have been associated with health benefits relevant to a number of disease conditions (*14*). Thus, berry fruits show antioxidant, anti-inflammatory, antiviral, antiproliferative, antimutagenic, antimicrobial, anticarcinogenic, antineurodegenerative, protection from cardiovascular damage and antiallergic effects (*15*).

Concerning the importance of consumption of berry fruit seeds with proven health-beneficial properties and the fact they can be obtained as a by-product from food processing companies, there is a need for complete phytochemical characterization among cultivars. The aim of the current work is to characterize seeds from 45 different genuine Serbian fruit cultivars by evaluation of their primary metabolites (sugars, lipids and fatty acids). The purpose of these evaluations is: (*i*) to raise the importance of the role of primary metabolites in chemotaxonomic classification of plants, and (*ii*) to provide a comprehensive comparative study of three major chromatographic techniques (gas chromatography coupled with mass spectrometry (GC-MS), high-performance thin-layer chromatography (HPTLC) and high-performance anion-exchange chromatography with pulsed amperometric detection (HPAEC-PAD)) for metabolomic profiling of cultivated berry fruit seeds. Two chemometric approaches, explorative analysis and classification, will be applied to separate useful information from noise, to provide visual inspection of multivariate data, and to uncover hidden correlations.

Considering a small role of primary metabolites in differentiation of plants in relation to their botanical and geographic origin, we expect that it should be possible that at least one class of these compounds can be used for authenticity assessment of seeds from berry fruits. Reliable biomarkers obtained from the primary metabolite profile could be a powerful tool in the authentication of botanical origin, and detection of natural product adulteration. Concerning berry fruit products, the knowledge on the primary metabolite composition obtained in this study may contribute to intercultivar genotypic and phenotypic discrimination among different cultivars, and could be useful for the development of new breeding technologies.

## MATERIALS AND METHODS

### Chemicals and materials

The fatty acid standard Supelco® 37 Component FAME Mix was purchased from Sigma-Aldrich, Merck (Burlington, MA, USA). Sugar standards: glucose (Glc), fructose (Fru), saccharose (Sac), trehalose (Tre), maltose (Mal) and arabinose (Ara) were purchased from TCI Europe N.V. (Zwijndrecht, Belgium), turanose (Tur), rhamnose (Rham), gentiobiose (Gent), isomaltose (Ism), panose (Pan), raffinose (Raf), isomaltotriose (Ismt), maltotriose (Malt), melibiose (Mel), xylose (Xyl), celobiose (Cel), melesitose (Mels), maltotriose (Maltotri), maltotetraose (Maltotetr), maltopentaose (Maltopen), maltohexaose (Maltohex) and maltoheptaose (Maltohep) were obtained from Tokyo Chemical Industry (TCI, Tokyo, Japan). Sorbitol (Sor), erythritol (Ert), arabinitol (Arabt), mannitol (Mant) and galactitol (Glt) were purchased from Sigma-Aldrich, Merck (Steinheim, Germany). Ultrapure water (MicroPure water purification system, 0.055 μS/cm, TKA, Thermo Fisher Scientific, Niederelbert, Germany) was used to prepare standard sugar solutions and blanks. Methanol, chloroform, diethylether, acetone and *n*-hexane were from Merck (KGaA, Darmstadt, Germany). Syringe filters (13 mm, polytetrafluoroethylene (PTFE) membrane 0.45 μm) were purchased from Supelco (Bellefonte, PA, USA). All reagents and chemicals whose purity was not previously emphased were of analytical purity grade.

### Plant material

**Fig. S1:**
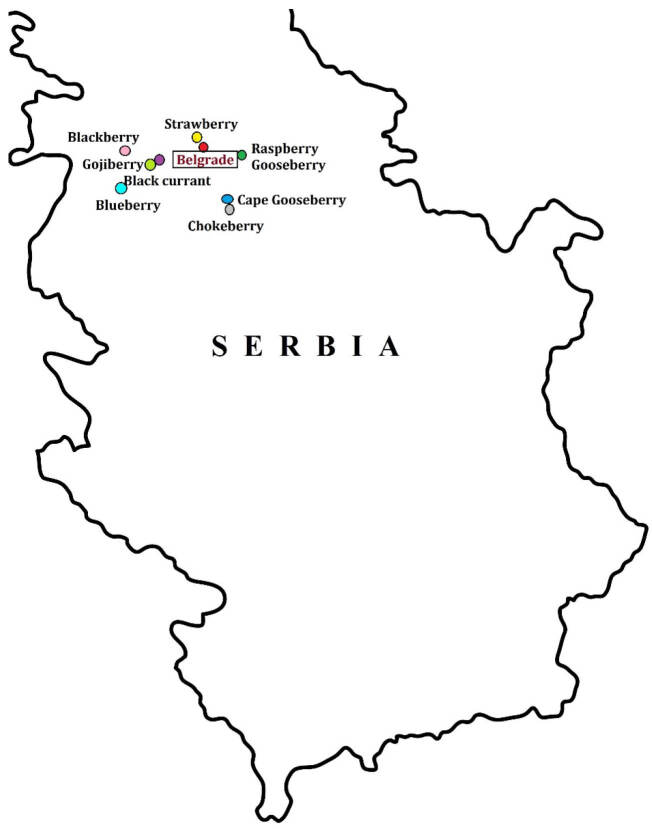
Map with marked locations of the orchards of berry fruits

Fruits from cultivars belonging to nine berry fruit species (strawberry, raspberry, blackberry, black currant, blueberry, gooseberry, chokeberry, cape gooseberry and goji berry) (Table 1) were picked in commercial orchards as presented in Fig. S1. The data were collected from a network of berry orchards with modern and intensive production system. The locations are situated in the middle of the north temperate zone, having continental climate with warm summers and cold winters. During the experimental year, the average daily temperature was between 11 and 11.5 °C. The average annual amount of precipitation in all locations was about 650 L/m^2^.

**Table 1:** List of analyzed berry cultivars

Sample number*	Species	Cultivar	Country of origin	GPS coordinates of fruit collection
Plate a	Strawberry			
1	*Fragaria* × *ananassa*	Sel.30.8	Italy	44°49'58.2"N 20°18'44.3"E
2		Capri	Italy	
3		Albion	USA	
4		Alba	Italy	
5		Premy	Italy	
6		Asia	Italy	
7		Joly	Italy	
8		Leatitia	Italy	
9		Garda	Italy	
10		Clery	Italy	
11		Roxana	Italy	
12		Brilla	Italy	
13		Jeny	Italy	
14		Irma	Italy	
15		VR 4	Italy	
16		Arosa	Italy	
Plate b	Blackberry			
1	*Rubus fruticosus*	Čačanska bestrina	Serbia	44°42'59.2"N 19°35'40.9"E
2		Loch Ness	Scotland	
3	*Rubus allegheniensis*	Triple crown	USA	
	Black currant			
4	*Ribes nigrum*	Ben Nevis	Scotland	44°39'00.0"N 20°14'15.4"E
5		Ben Sarek	Scotland	
6		Bona	Poland	
7		Čačak black	Serbia	
8		Malling Juel	England	
9		Ojebin	Sweden	
10		Ometa	Switzerland	
11		Silmu	The Netherlands	
12		Tenah	The Netherlands	
13		Titania	Sweden	
14		Triton	Sweden	
15		Tsema	The Netherlands	
Plate c	Raspberry			
1	*Rubus idaeus*	Glen Ample	Scotland	44°45'11.4"N 20°35'08.0"E
2		Meeker	USA	
3		Tulameen	Canada	
4		Willamette	USA	
	Gooseberry			
5	*Ribes uva-crispa*	Hinnonmaki red	Finland	44°45'11.4"N 20°35'08.0"E
6		Hinnonmaki yellow	Finland	
	Chokeberry (aronia)			
7	*Aronia arbutifolia*	Nero	Czech Republic	44°10'10.8"N 20°28'44.0"E
	Goji berry			
8	*Lycium barbarum*	Ningxia No 1	China	44°37'11.3"N 20°06'30.2"E
	Cape gooseberry			
9	*Physalis peruviana*	Goldenberry	Peru	44°11'35.3"N 20°16'44.3"E
	Blueberry			
10	*Vaccinium corymbosum*	Bluecrop	USA	44°26'05.4"N 19°53'17.0"E
11		Brigita blue	Australia	
12		Duke	USA	
13		Patriot	USA	
14		Spartan	USA	

**Fig. 1:**
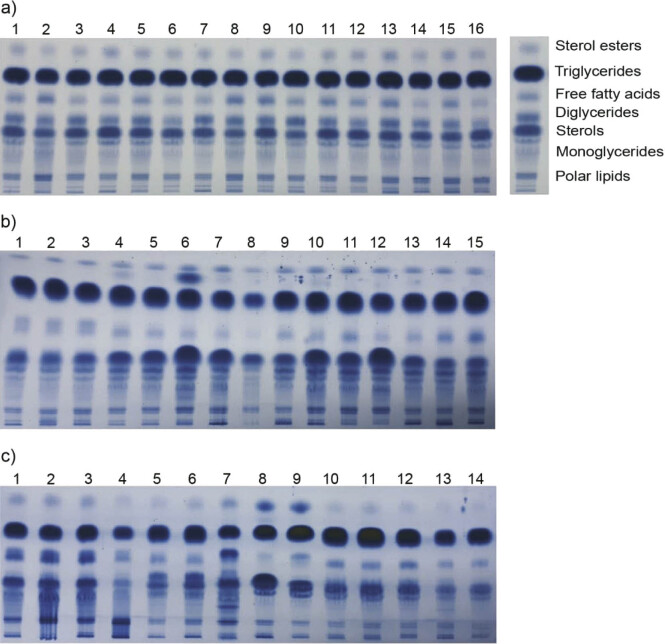
High-performance thin-layer chromatograms of lipids in berry seeds of: a) strawberry, b) blackberry (1–3) and black currant (4–15), and c) raspberry (1–4), gooseberry (5 and 6), chokeberry (7), goji berry (8), cape gooseberry (9) and blueberry (10–14) cultivars

*Sample numbers correspond to the chromatograms in Fig. 1

Before planting, soil was prepared, weed-controlled, and pH was measured. Planting distance and support was adapted to each fruit species/cultivar. All necessary agrotechnical measurements, such as pruning, fertilization and irrigation were done regularly, while weeding and pest control were done according to the Integrated pest management directive 2009/128/EC (*16*). All nursery plants used for planting orchards were purchased from certified nurseries who guaranteed that plant material was propagated from a known nucleus plant with inspected phytosanitary status, claiming that it is true to varietal type (accompanied with original tag).

Strawberry orchard was organized on raised double beds covered with black polyethylene foil. Blueberries were planted at raised beds mulched with agrotextile. Plots with raspberry, blackberry, currants, gooseberry, chokeberry, goji berry and cape gooseberry were prepared by clean tillage, where the space between plants, within the row, was hand weeded. All orchards had a system for drip irrigation which was used for fertigation too. In the spring, plants were fertilized with NPK fertilizer (20:20:20) to stimulate vegetative growth, while formulation NPK 11:11:33 was utilized in the cropping stage. For strawberries, foliar application of Ca fertilizers with microelements and amino acids was applied too. For blueberries, ammonium, potassium and magnesium sulphate were added in the phase of fruit setting and fruit development.

At the harvesting time, a sample of 50 fully ripened fruits was collected randomly from all around five plants/bushes (5 plants×10 fruits). Berries that were picked had typical shape and colour, were undamaged and without any signs of diseases. Seeds were manually removed from mesocarp just after picking, rinsed with tap water, air dried in the dark place for 10 days at room temperature (20 °C) and stored in paper bags until the analysis.

### Extraction procedures

#### Extraction of lipids

Before extraction, samples of berry seeds were ground in a mill with liquid nitrogen (A 11 basic analytical mill; IKA-Werke GmbH & CO. KG, Staufen, Germany) to obtain a fine powder. Lipids were extracted from fruit seed samples according to the Folch method (*17*). Approximately 2 g of seed samples were extracted with a mixture of *V*(CHCl_3_):*V*(MeOH)=2:1 (90 mL) using an ultrasonic bath (Sonic, Niš, Serbia) for 6 h, and after that centrifuged at 7000×*g* for 10 min (centrifuge SL 16; Thermo Fisher Scientific, Waltham, MA, USA). Extractions were performed in duplicate. The organic phases were collected and washed with 0.88% KCl aqueous solution and mixture of *V*(MeOH):*V*(H_2_O)=1:1. The obtained lipid fraction was dried over anhydrous MgSO_4_, filtered and evaporated to dryness under reduced pressure with a rotary evaporator (IKA RV 05 basic rotary evaporator; IKA-Werke). The dried seed oil was dissolved in 4 mL of chloroform and stored at -20 °C.

#### Preparation of FAMEs

Fatty acid methyl esters (FAME) were obtained from the extracted oil using transmethylation under alkaline conditions according to ISO 12966-2:2017 (*18*). Extracted oil (approx. 0.1 g) was dissolved in 2 mL *n*-hexane and then 1 mL of 2 mol/L methanolic potassium hydroxide solution was added and vortexed (basic vortex mixer; Thermo Fisher Scientific) for 2 min at room temperature. Hexane phase was separated after centrifugation at 4000×*g* (centrifuge SL 16; Thermo Fisher Scientific, Dreieich, Germany) for 5 min and neutralized by adding 1 g of sodium hydrogen sulphate monohydrate. After salt precipitation, upper fraction was transferred to a vial for further FAME analysis.

#### Extraction of carbohydrates

Samples (0.1 g of each) were dissolved in 10 mL ultrapure water and the solution was mixed for 30 min in an ultrasonic bath (frequency 40 kHz, voltage 220 V, produced by Sonic) at room temperature (20 °C). After centrifugation at 8000×*g* (centrifuge SL 16; Thermo Fisher Scientific) for 10 min, the solution was filtered through 0.45-μm PTFE membrane filter and carbohydrate content was determined.

### High-performance thin-layer chromatography analysis

Aliquots of 1 µL of each seed lipid extract were applied to the 20 cm×10 cm HPTLC silica gel plates (Merck, Darmstadt, Germany) as 6-mm band by using Automatic TLC sampler 4 (ATS4; CAMAG, Muttenz, Switzerland). Plates were developed in the saturated twin trough chamber up to a migration distance of 90 mm, using four mobile phases: *V*(chloroform):*V*(methanol):*V*(acetic acid)=90:10:1 up to 25 mm, *V*(*n*-hexane):*V*(diethylether):*V*(acetone)=60:40:5 up to 70 mm, *V*(*n*-hexane):*V*(diethylether)=97:3 up to 85 mm and 100% *n*-hexane up to 90 mm. The developed plates were dried in a stream of warm air and dipped in post-chromatographic derivatization solution of cerium ammonium molybdate for 1 s with an immersion speed of 3.5 cm/s, using Chromatogram Immersion Device III (CAMAG). Derivatized plates were heated for 5 min at 110 °C on TLC Plate Heater III (CAMAG). Images were captured at 366 nm with DigiStore 2 device image analysing system in conjunction with Reprostar 3 (CAMAG). Four apertures with exposure time of 30 ms and frame of 2 mm were applied. The photos were stored as TIF files for further image processing.

### Gas chromatography-mass spectrometry

Gas chromatography-mass spectrometry (GC-MS) analysis was performed using a Thermo Fisher Focus GC coupled with Polaris Q system (Carlsbad, CA, USA). The column was Thermo Scientific’s TRACE™ TR-WaxMS (30 m×0.25 mm×0.25 µm). Oven temperature program was as follows: initial temperature 50 °C for 1 min, then increased to 200 °C at 25 °C/min and finally to 230 °C at 3 °C/min for 18 min.

Injector, transfer line and ion source temperatures were 250, 260 and 260 °C, respectively. Injection volume was 1 μL and the injector was in split mode (50:1). Helium with a flow rate of 1 mL/min was used as the carrier gas. The analysis time was 35 min. Fatty acid methyl esters were identified by comparison of the retention times with previously injected mixture of the standards and the resulting mass spectra to those of injected standards and those given in the NIST database (*19*). Results are presented as relative content (%) obtained after integration of the total ion chromatograms.

### High-performance anion-exchange chromatography with pulsed amperometric detection

Carbohydrate analysis was performed on an ICS3000 DP liquid chromatography system (Dionex, Sunnyvale, CA, USA) equipped with a quaternary gradient pump (Dionex). The carbohydrates were separated on a CarboPac® PA100 pellicular anion-exchange column (4 mm×250 mm; Dionex) at 30 °C. An ICS AS-DV 50 autosampler (Dionex) was used for sample injection (injection volume for each sample was 25 μL). The mobile phase, consisting of 600 mM sodium hydroxide (A), 500 mM sodium acetate (B) and ultrapure water (C), was used according to the following linear gradient (flow rate, 0.7 mL/min): 0–5 min, 15% A, 85% C; 5.0–5.1 min, 15% A, 2% B, 83% C; 5.1–12.0 min, 15% A, 2% B, 83% C; 12.0–12.1 min, 15% A, 4% B, 81% C; 12.1–20.0 min 15% A, 4% B, 81% C; 20.0–20.1 min 20% A, 20% B, 60% C; 20.1–30.0 min 20% A, 20% B, 60% C. Before the analysis, the system was preconditioned with 15% A and 85% C for 15 min. The pulsed amperometric detector consisted of gold as the working electrode and Ag/AgCl as the reference one. Data were collected using Chromeleon software (*20*) and exported to Excel for further analysis.

### Data acquisition and statistical analysis

Images of the HPTLC chromatograms were processed with the ImageJ software (*21*). Before the signal acquisition and analysis, each image was split-filtered through red, green and blue channel. Denoising of filtered images was done using median filter with width of 2 pixels. Bandpass filter, which filters large structures down to 40 pixels and small structures up to 3 pixels, was used for removing the differences of the background intensity between images. Chromatographic signals were extracted from images in form of pixel intensity line profiles.

Pretreatment of chromatographic signals (HPTLC, GC-MS and HPAEC-PAD) consisted of warping, normalization and centring, and was performed by algorithms implemented in the PLS ToolBox software for MATLAB (*22*). Warping procedure was performed due to the spatial differences of the band locations. Two variable alignment algorithms were applied: correlation optimized warping (COW) and peak alignment. Normalization step was used to adjust the values measured on different scales to a notionally common scale. Weighted normalization of the chromatograms was done by standard normal variate (SNV) transformation. The data were additionally pre-processed by mean or median centring, which is the preferred option when the classification of samples is based on variables that are all measured in the same unit.

Principal component analysis (PCA) and partial least square-discriminant analysis (PLS-DA) were performed by PLS ToolBox (*22*). PCA was carried out by using a singular value decomposition algorithm and a 0.95 confidence level for Q and T2 Hotelling limits for outliers. The PLS-DA was performed using the SIMPLS algorithm without forcing orthogonal conditions to the model in order to condense Y-block variance into first latent variables. The optimal complexity and reliability of the resulting models was ensured by fourfold cross-validation (CV) which employed Venetian blinds resampling strategy. In order to assess predictive performance of PLS-DA models, *i.e*. to determine the degree of accuracy and sensitivity, the entire data set of 45 samples was divided into two subsets, a model building and optimization set consisting of 33 randomly selected objects, and testing set composed of 11 objects. The same subsets of berry samples were used for all chromatographic methods. The quality of the models was monitored with the following parameters: (*i*) R^2^_cal_, the cumulative sum of squares of the Ys explained by all extracted components, R^2^_CV_, the cumulative fraction of the total variation of the Ys that can be predicted by all extracted components, and R^2^_PRED_, the cumulative fraction of the total variation of the Ys that can be predicted by test components, and (*ii*) root mean square errors of calibration (RMSEC), root mean square errors of cross-validation (RMSECV) and root mean square errors of prediction (RMSEP).

## RESULTS AND DISCUSSION

### Primary metabolite profiles

#### Lipid profiling

Complete lipid characterization of analysed berry seed cultivars was performed by HPTLC fingerprinting of the main lipid classes (polar lipids, monoacylglycerols, sterols, diacylglycerols, free fatty acids, triacylglycerols and sterol esters, retention factor *R*_F_≈0.11, 0.22‒0.32, 0.42, 0.48, 0.62, 0.75 and 0.92, respectively, Fig. 1) in order to screen the contribution of those involved in fundamental metabolic processes (free fatty acids and acylglycerols). Due to the complex composition of berry seed extracts, optimization of HPTLC conditions was done regarding the resolution of the low, medium and high polarity lipid compounds. Optimal results with vast number of sharp bands, maximum separation efficiency and lowest background noise for all analysed species and all lipid classes were obtained using multiple one-dimensional chromatographic systems (listed in the experimental part). The zones on the chromatograms were identified based on the knowledge of the polarity of each lipid class, as well as by comparison with the previously published chromatograms (*23*).

**Fig. S2:**
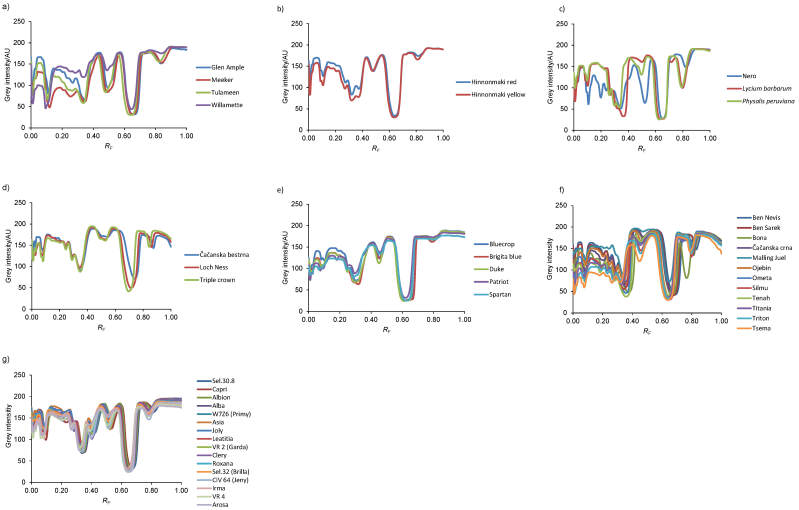
Chromatograms adjusted to gray scale for: a) raspberry, b) gooseberry, c) chokeberry, goji berry and cape gooseberry, d) blackberry, e) blueberry, f) black currant, and g) strawberry

Visual examination of the HPTLC chromatograms (Fig. 1) and the corresponding plots of chromatographic signals (Fig. S2) indicates the uniformity of lipid profiles within each fruit species, but also reveals differences in the chemical composition depending on the botanical origin. The same lipid classes are present in all samples, but different compounds are present in various amounts within each class. Therefore, the presence or absence of zones in a particular group of samples was noted, as well as the variability of the signal intensity of the compounds at the same *R*_F_ values​. The chromatograms of the strawberry samples showed high intensity zones or peaks at *R*_F_=0.75 that correspond to triglycerides and well separated medium intensity zones at *R*_F_=0.62 that correspond to free fatty acids (Fig. 1a, samples 1–16). Black currant samples (Fig. 1b, samples 4–15, pixel intensity 1846) and raspberries (Fig. 1c, samples 1–4, pixel intensity 2008) have more intense and characteristic zones at lower *R*_F_ values between 0.1 and 0.3 which are considered as polar lipids. Raspberry seed samples (Fig. 1c, samples 1-4) have also intense zones that correspond to free fatty acids (*R*_F_=0.62, pixel intensity 6246). The zones at the highest *R*_F_ values (0.92) that correspond to sterol esters are the most intense in the goji and cape gooseberry samples (Fig. 1c, samples 8 and 9, intensity of pixels 5660 and 6240, respectively), while the same zones are hardly noticeable in the blueberry samples (Fig. 1c, samples 10–14, 973 pixels). The chromatogram obtained for chokeberry seed extract (Fig. 1c, band 7) has more zones than the rest of the analysed samples. The chromatogram profiles of goji and cape gooseberry lipids (Fig. 1c, bands 8 and 9) are similar to raspberry samples (Fig. 1c, bands 1–4), while the gooseberry (Fig. 1c, bands 5 and 6) are similar to blueberries (Fig. 1c, bands 10–14).

#### Fatty acid profiling

**Fig. 2:**
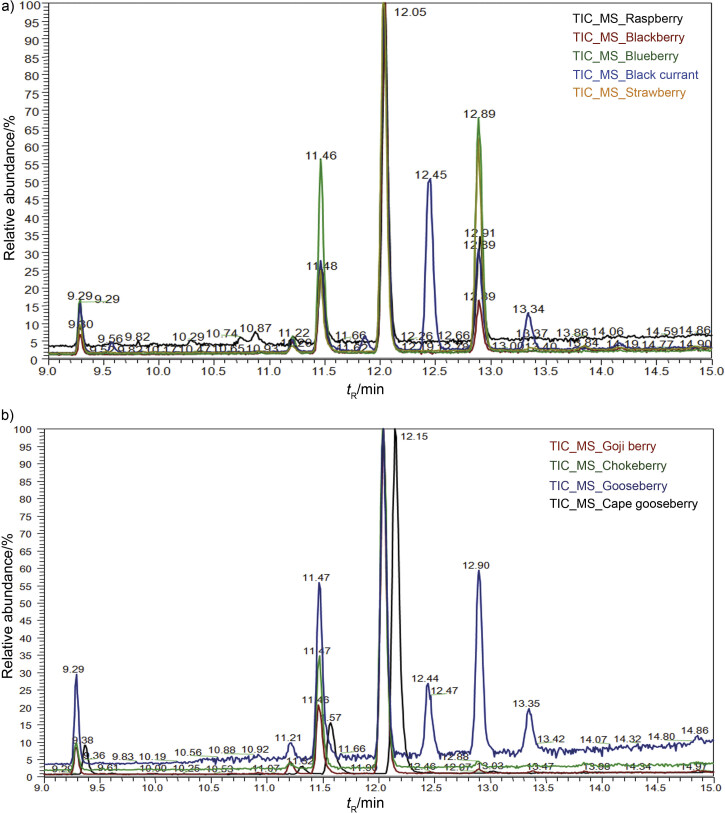
Fatty acid methyl ester chromatograms of analyzed berry species: a) strawberry, black currant, blueberry, blackberry and raspberry cultivars, and b) cape gooseberry, goji berry, chokeberry and gooseberry cultivars

Qualitative description of differences between fatty acid profiles of plant species is based on the presence and intensity of the fatty acid signals (Fig. 2 and Table S1). Fatty acid profiles within the same fruit species showed specific pattern, but different compared to other species. The most abundant fatty acid in the analysed berry seed oils was linoleic acid (*t*_R_=12.07 min, 44.36‒80.09%), followed by oleic (*t*_R_=11.48 min, 12.71‒26.38%) and α-linolenic (*t*_R_=12.90 min, 0.53‒31.42%) acids (Table S1). Oomah and Ladet (*24*) previously reported that 96% of the total fatty acid composition of raspberry seed oil consists of these three fatty acids. Linoleic acid was the most prevalent in cape gooseberry and goji berry samples, oleic acid in gooseberry, blackberry, chokeberry and blueberry samples, and α-linolenic in strawberry and blueberry seeds (Fig. 2a). Literature data (*9*, *25*) confirmed low content of α-linolenic acid in goji (1.1%) and chokeberry (0.34%) fruit, similar to samples of goji, gooseberry, chokeberry and cape gooseberry (0.53‒1.41%) analysed in this study (Fig. 2b). Parker *et al.* (*26*) showed that cranberry seed oil contains about 22% α-linolenic acid, indicating that the fruit seeds may be a potential dietary source of this acid.

Higher abundance of γ-linolenic acid (*t*_R_=12.46 min) was noticed in black currant and gooseberry samples (14.63 and 12.26%, respectively) (Table S1) confirming that seed oil from Grossulariaceae (genus *Ribes*) family are rich in γ-linolenic acid (from 18.3% in gooseberry to 30.5% in black currant) (*27*). On the contrary, it was not found in strawberry, raspberry and blackberry seed oil, a typical feature of the Rosaceae family. This is an indicator of the differences in fatty acid desaturase profiles of Rosaceae and Grossulariaceae (*Ribes*) species within the same superorder Rosanae (*11*).

Only in the samples of black currant *cis*-11-eicosenoic acid was found (*t*_R_=14.19 min, 2.46%). Heneicosapentaenoic acid (21:5, n−3) (*t*_R_=13.34 min) is an unsaturated fatty acid that has been identified in gooseberry and black currant samples, by comparison of the resulting mass spectra to the NIST database (*19*), as well as 6-octadecanoic acid (*t*_R_=11.87 min) detected only in black currant.

All analysed berry seeds contained low amounts of saturated fatty acids, namely palmitic (*t*_R_=9.30 min, 3.07‒8.56%) and stearic (*t*_R_=11.23 min, 1.14‒11.47%) acids (Table S1). Palmitic acid was the major saturated fatty acid detected in gooseberry seeds and pulp by Ramadan and Mörsel (*28*). Arachidic acid (*t*_R_=13.86 min) showed weak signals present only in strawberry, blackberry and goji berry seed samples (0.50‒0.77%) (Table S1).

#### Sugar profiling

Twenty-eight sugars were identified, including five monosaccarides, eight disaccharides, five trisaccharides, and one tetra-, penta-, hexa- and heptasaccharide, as well as six sugar alcohols (Table S2). The most prevalent sugars in all analysed cultivars were Glu (*t*_R_=5.91 min, average mass fraction 61.67‒148.60 mg/kg), Fru (*t*_R_=6.88 min, 67.17‒173.21 mg/kg) and Sac (*t*_R_=9.70 min, 91.17‒162.16 mg/kg) (Table S2). Trehalose, Mel, Tur, Raf, Pan, Maltotri, Maltotetr, Maltopen, Maltohex and Maltohep were present as medium intensity signals (Table S2). Among these sugars, Pan had the highest intensity peaks in all berry species, particularly in blackberry seeds (*t*_R_=18.11 min, 5.91‒55.41 mg/kg), which is in accordance with previous studies of sugar mass fraction in apricot kernels (*29*). Maltose, Ara, Rham, Gent, Ism, Ismt, Xyl, Cel and Mels occurred as minor constituents at different mass fractions (Table S2), produced as responses to various environmental influences.

Glycerol was the most abundant sugar alcohol in all seed samples (*t*_R_=2.36 min, 9.03‒29.48 mg/kg) because seeds represent a storage of lipids in plants mostly in the form of triacylglycerols (formed by the esterification of glycerol and the three fatty acids), which easily hydrolyzed under the conditions of sugar analysis (alkaline medium). Other analyzed sugar alcohols showed medium (Ert and Sor) or low intensity signals (Arabt, mannitol and Glt) (Table S2).

### Primary metabolites in chemotaxonomic classification

In order to explore a multivariate space of chromatographic profiles and look for possible patterns between samples and chromatographic signals, PCA was applied on each data set separately. The matrix consisted of 45 objects (tested samples), 1200 variables representing the intensities of pixels along the 1200 length lines obtained by digitization of lipid chromatograms (using green channel of profiles as the most informative one), 2384 variables representing intensities of analytical signals on fatty acid chromatograms, and 1797 variables representing intensities of analytical signals on sugar chromatograms.

**Fig. 3:**
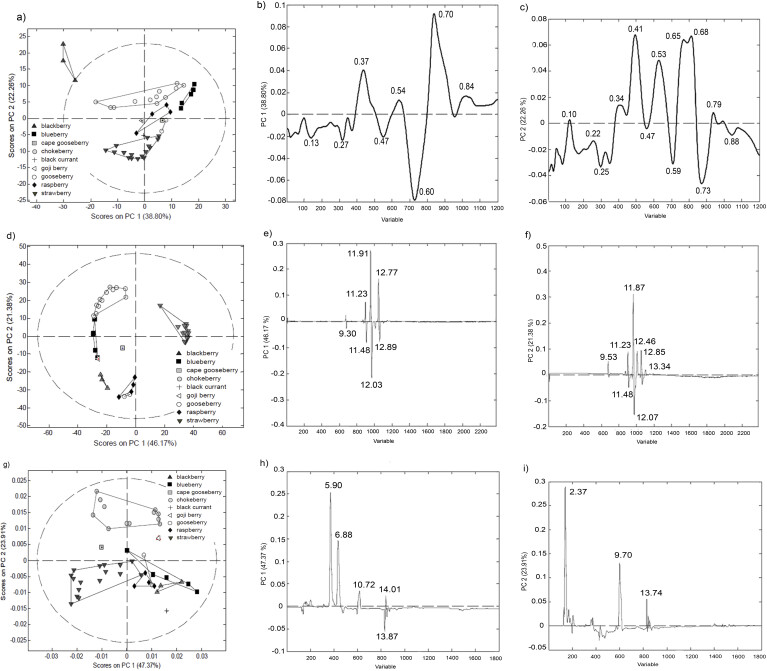
Principal component analysis: a) score plot, b and c) loading plots for lipid profile, d) score plot, e and f) loading plots for fatty acid profile, g) score plot, and h and i) loading plots for sugar profile of seeds from berry fruit cultivars. Number of 1200 variables represent the intensities of pixels along the 1200 length lines obtained by digitization of lipid chromatograms (using green channel of profiles as the most informative one), 2384 variables represent intensities of analytical signals on fatty acid chromatograms, and 1797 variables represent intensities of analytical signals on sugar chromatograms. Intensities of signals were transformed in *R*_F_ and *t*_R_ scale in order to determine the most influential variables

All PCA models are graphically presented as mutual projections of factor scores and their loadings for the first two principal components (PCs) (Fig. 3).

#### Lipids as markers of botanical origin

The obtained four-component PCA model explained 77.49% of total variance, PC1 accounted for 38.80% of the overall data variance and PC2 for 22.26%. The score plot revealed the existence of five clusters corresponding to different berry fruit cultivars (blackberry, strawberry, black currant, raspberry and blueberry) (Fig. 3a). A small number of samples of gooseberry, cape gooseberry, chokeberry and goji berry interrupted their complete differentiation, but even so, PCA model suggested similar lipid profile of these cultivars to those of raspberry and strawberry (Fig. 3a).

The zones with *R*_F_ values 0.37 and 0.54 (corresponding to free fatty acids), 0.70 (corresponding to triglycerides) and 0.84 with the greatest positive effects on PC1 axis and the zones with *R*_F_ values 0.13, 0.27, 0.47 and 0.60 with the greatest negative effect have the strongest contribution to the positioning of the objects on the score plot (Fig. 3b). Additionally, zones with *R*_F_ values 0.34 (corresponding to sterol esters), 0.41 (corresponding to diglycerides), 0.53 (corresponding to free fatty acids), 0.65 and 0.68 (corresponding to triglycerides) and 0.79 (corresponding to sterol esters) significantly affect the PC2 in a positive manner, while the zones with negative influence on PC2 have *R*_F_ values 0.22, 0.25, 0.41, 0.53, 0.59 and 0.73 (Fig. 3c).

The content of free fatty acids and triglycerides significantly affects the differentiation of blueberry and gooseberry seeds, while sterol esters, diglycerides, triglycerides and free fatty acids were major factors that separate the blueberry, black currant and blackberry samples.

#### Fatty acids as markers of botanical origin

The obtained four-component PCA model explained 84.96% of total variance, PC1 accounted for 46.17% of the overall data variance and PC2 for 21.38%. The score plot indicated differentiation of samples according to botanical origin (Fig. 3d). Blackberry, strawberry, black currant, raspberry and blueberry seed samples formed five compact clusters, suggesting uniform free acid profiles within certain cultivar. Gooseberry overlaps with raspberry, while goji berry and chokeberry overlap with blueberry seed samples (Fig. 3d). Although black currant and gooseberry samples show specific signals on chromatograms at *t*_R_=12.45 and 13.34 min, which correspond to γ-linolenic and heneicosapentaenoic acids, respectively (Fig. 2, Table S1), they are differently classified according to their whole fatty acid profiles. Cape gooseberry seed sample is separated from other berry cultivars (Fig. 3d).

Loading plot revealed that signals at *t*_R_=9.30, 11.48, 12.03 and 12.89 min (corresponding to palmitic, oleic, linoleic and α-linolenic acids, respectively) had the highest negative effect on PC1 component, while variables at *t*_R_=11.23 (stearic acid), 11.91 and 12.77 min had the highest positive effects (Fig. 3e). Hence, these variables had the highest influence on the differentiation of strawberry cultivars from others. Signals that potentially have the highest influence on sample separation along PC2 were recorded at *t*_R_=9.53 (palmitoleic acid), 11.23, 11.48, 11.87 (6-octadecanoic acid), 12.07, 12.46 (γ-linolenic acid), 12.90 and 13.34 min (Fig. 3f). Among the identified fatty acids, those corresponding to retention times 9.53, 11.87, 12.46 and 13.34 min are considered as specific markers of black currant seed. Additionally, signal at 11.23 min defines blueberry samples. However, since the score plot revealed differentiation of the analysed berry cultivars, the whole fatty acid profile must be observed for characterization of certain botanical origin.

#### Sugars as markers of botanical origin

The obtained four-component PCA model explained 82.18% of total data variance, out of which PC1 accounted for 47.37%, and PC2 for 23.91%. Contrary to the results obtained from fatty acid and lipid chromatographic fingerprints, PCA of sugar fingerprints (Fig. 3g) revealed differentiation of black currant and strawberry seed samples, while blackberry, raspberry and blueberry seed formed partially overlapping groups. Cape gooseberry, chokeberry and goji berry seed samples showed unique sugar profiles, different from other cultivars (Fig. 3g).

Loading plot (Fig. 3h) revealed that variables with the highest positive impact on separation along PC1 direction were signals at *t*_R_=5.90 (Glu), 6.88 (Fru), 10.72 (Cel) and 14.01 min (Gent). The most influential parameters along PC2 (Fig. 3i) axis were sugar signals at *t*_R_=2.37 (glycerol), 9.70 (Sac) and 13.74 min (Mels).

### Comparative study of chromatographic approaches for metabolite profiling and sample classification

Comparison of the suitability of the studied chromatographic methods for chemotaxonomic classification of seeds from cultivated berry fruits was analysed based on the statistical parameters of PLS-DA classification models. Given the limited number of botanical species with satisfactory number of samples, only black currant, strawberry, blueberry and raspberry seeds were evaluated.

**Fig. S3:**
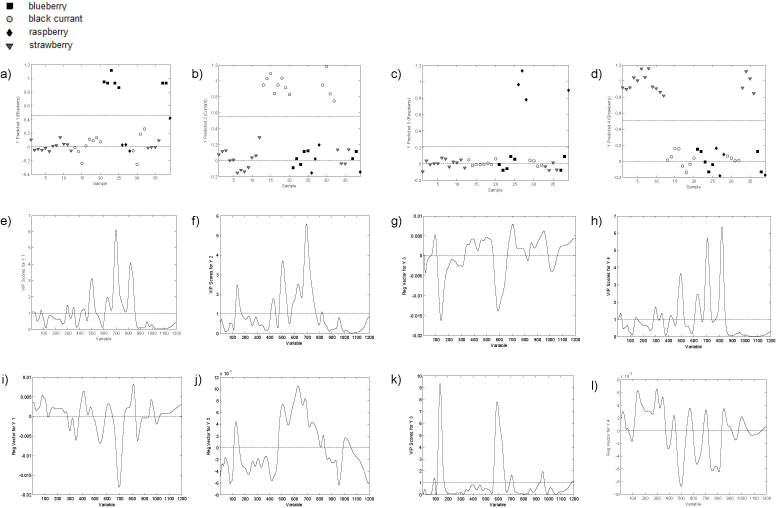
Partial least square-discriminant analysis of lipid profiles of berry seeds: a-d) score plots of data, e-h) plots of the variables (intensity of pixels) *versus* variable importance in the projection scores, i-l) plot of the coefficients of parameters

**Fig. S4:**
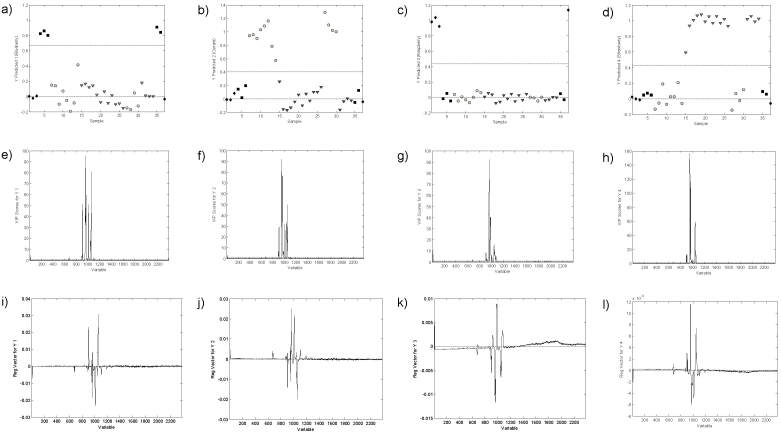
Partial least square-discriminant analysis of fatty acid profiles of berry seeds: a-d) score plots of data, e-h) plots of the variables (intensities of analytical signals) *versus* variable importance in the projection scores, i-l) plot of the coefficients of parameters

**Fig. S5:**
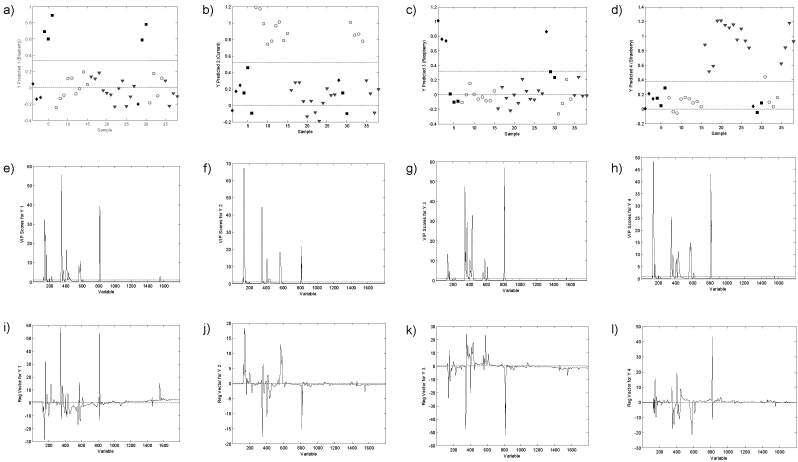
Partial least square-discriminant analysis of sugar profiles of berry seeds: a-d) score plots of data, e-h) plots of the variables (intensities of analytical signals) *versus* variable importance in the projection scores, i-l) plot of the coefficients of parameters

All classification models demonstrate good statistical performance (Table 2, Fig. S3, Fig. S4, Fig. S5), with the coefficient of determination ranging from R^2^=0.4734, for the blueberry seeds based on HPAEC sugar profiles, to R^2^=0.9940, for the raspberry seeds and GC-MS profiles of fatty acids. Although the best chemotaxonomic classification was achieved based on the GC-MS data, with average R^2^=0.9058 and 0% misclassification error (Table 2), it is closely followed by PLS-DA models based on HPTLC profiles of lipids (with R^2^ in the range of 0.7171–0.958). However, mathematical model based on HPAEC profiles of sugar components was slightly worse (R^2^ ranging from 0.4734 to 0.8801, with arithmetic mean 0.7392). We believe that this is not a result of shortcomings of HAEC-PAD technique, but probably a result of huge variations of sugar content, even within each particular cultivar, which further prevented the best differentiation of classes due to the overlapping ranges.

**Table 2:** Statistical performances of the partial least square-discriminant analysis (PLS-DA) models for classification of four groups of berry fruit seeds, based on HPTLC (lipids), GC-MS (fatty acids) and HPAEC-PAD (sugars) chromatographic profiles

Parameter	Lipid profile				Fatty acid profile				Sugar profile			
Blueberry	Black currant	Raspberry	Strawberry	Blueberry	Black currant	Raspberry	Strawberry	Blueberry	Black currant	Raspberry	Strawberry
R^2^_cal_	0.9434	0.9270	0.8912	0.9316	0.8061	0.9000	0.9786	0.9509	0.7771	0.8604	0.8774	0.8737
R^2^_CV_	0.8762	0.8710	0.7171	0.8489	0.7034	0.8067	0.9452	0.9021	0.4734	0.7861	0.4935	0.5395
R^2^_PRED_	0.7937	0.9052	0.8872	0.9582	0.9318	0.9717	0.9940	0.9789	0.8219	0.8801	0.6537	0.8332
RMSEC	0.0911	0.1221	0.1020	0.1294	0.0911	0.1221	0.1020	0.1294	0.1569	0.1826	0.1176	0.1819
RMSECV	0.1348	0.1664	0.1668	0.1939	0.1348	0.1664	0.1668	0.1939	0.2544	0.2221	0.2444	0.3654
RMSEP	0.1835	0.1502	0.1057	0.1020	0.1103	0.1148	0.0493	0.0738	0.1961	0.1834	0.1813	0.2012
Class. err. Cal/%	0.00	0.00	0.00	0.00	0.00	0.00	0.00	0.00	0.00	0.00	0.00	0.00
Class. err. CV/%	0.00	9.03	0.00	4.17	0.00	0.00	0.00	0.00	0.00	2.78	1.67	10.00
Class. err. Pred/%	0.00	0.00	0.00	0.00	0.00	0.00	0.00	0.00	0.00	0.00	0.00	7.14

R^2^_cal_=the cumulative sum of squares of the Ys explained by all extracted components, R^2^_CV_=the cumulative fraction of the total variation of the Ys that can be predicted by all extracted components, R^2^_PRED_=the cumulative fraction of the total variation of the Ys that can be predicted by test components, RMSEC=root mean square errors of calibration, RMSECV=root mean square errors of cross-validation, RMSEP=root mean square errors of prediction, HPTLC=high-performance thin-layer chromatography, GC-MS=gas chromatography-mass spectrometry, HPAEC-PAD=high-performance anion-exchange chromatography with pulsed amperometric detection

Although GC-MS provides the best overall performance of PLS-DA models, these models are slightly better for the separation of strawberry and raspberry seeds from the rest (averaged calibration, cross-validation and prediction R^2^ values for these two classes are 0.9440, and 0.9726, respectively), while in the case of black currant and blueberry seeds, the HPTLC profiles result in PLS-DA models with slightly better separation/statistical parameters (averaged R^2^ values are 0.9011 and 0.8350, respectively).

Such good results of GC-MS, in terms of providing the most suitable chromatographic fingerprints for PLS-DA models, go along with the fact that MS coupled to HPLC and GC is the most widely used analytical tool for profiling highly complex mixtures of primary metabolites. However, GC-MS analysis requires extensive sample preparation and derivatization procedures, while the retention of primary metabolites in the mostly used reversed-phase LC mode is very low (*2*). Despite continuous development of these sophisticated techniques, such multianalyte methods require fine tuning of a multitude of parameters, they need highly trained analysts and suffer from high costs of equipment and reagents. Therefore, the HPTLC, which gives equally suitable classification models, can be a method of choice for the first stage of analysis in generation of data for biomarker discovery. The main advantages of HPTLC method is its simplicity, flexibility, accessibility and low cost. With development of high-performance adsorbent layers and sophisticated instrumentation for sample application, chromatogram development, derivatization and chromatogram evaluation, its employment in fingerprint analysis is significant, as this study also confirms. Additionally, the main advantage of HPTLC method in the analysis of primary metabolites is a possibility of application of wide range of solvents of different polarity combined in multi-one-dimensional chromatography system.

Having in mind that all PLS-DA models resulted in statistically sound chemotaxonomic classification of berry seeds, it is clear that an approach based on combining advanced data analysis with non-targeted chromatographic profiling was able to overcome the principal challenges associated with untargeted metabolite profiling (*30*). First drawback of the untargeted strategy, which comprises complex protocols and time required to reduce the extensive datasets generated from chromatographic profiles into smaller set of manageable signals, was overcome by the application of simple dimensionality reduction to few latent variables (PCA and PLS-DA). The difficulties in identifying and characterizing unknown metabolites, as a second drawback of the untargeted strategy, were solved by using the entire chromatographic signal as a unique chemical fingerprint of the extract, without identification of individual peaks. The third drawback of the untargeted strategy is the bias towards detection of highly abundant metabolites. Reducing the dominance of highly abundant signals over those present in smaller quantity has been achieved by adequately processing the data prior to multivariate analysis.

## CONCLUSIONS

Metabolic approach which includes chromatographic profiling, data preprocessing and chemometrics was proposed to reveal differences in the composition of primary metabolites among seeds of different berry fruit cultivars. Blackberry, strawberry, black currant, raspberry and blueberry seed samples were differentiated according to lipid (di- and triglycerides, and sterol esters) and free acid profiles, while only black currant and strawberry seed samples showed specific sugar profiles. Unknown molecules that establish a characteristic chemical pattern of extracts were included in the statistical analysis-based fingerprint methodology and proved to be valuable parameters for consistent and accurate authenticity control.

Mathematical models for three applied techniques indicate equal opportunities of HPTLC and GC-MS for authenticity assessments of berry seeds. However, due to its simplicity, flexibility, accessibility and cheapness, HPTLC could be a method of choice for the first stage of analysis in generation of data for biomarker discovery.

The knowledge of metabolite composition obtained in this study can provide a better assessment of genotypic and phenotypic differences among berry fruit species and varieties, and could contribute to the development of new breeding programs. However, further investigation should consider observation of the influence of environmental factors on the chemical profile of berry seeds.

## Appendix

**Table S1:** Relative content of fatty acid methyl esters (FAMEs) detected in the berry seed samples and their retention times (*t*_R_)

*t*_R_/min	FAME carbon number	Systematic name	Common name	(*A*_seed_/*A*_total_)/%								
Strawberry	Blackberry	Black currant	Raspberry	Gooseberry	Chokeberry	Goji berry	Cape gooseberry	Blueberry
9.30	C16:0	methyl hexadecanoate	methyl palmitate	3.17	3.33	5.18	3.07	8.56	3.87	4.43	4.86	3.83
9.53	C16:1	methyl* cis*-9-hexadecanoate	methyl palmitoleate	0.22	-	0.41	-	-	-	0.28	0.16	0.11
11.23	C18:0	methyl octadecanoate	methyl stearate	1.14	2.28	1.73	1.17	2.95	1.68	2.46	1.65	11.47
11.48	C18:1 (*c*+*t*)	methyl* cis*-and *trans*-9-octadecanoate	methyl oleate and methyl elaidate	13.33	22.93	13.63	15.64	26.38	22.78	15.52	12.71	21.81
12.07	C18:2n-6 (*c*+*t*)	methyl* cis*- and *trans*-9,12-octadecadienoate	methyl linoleate and methyl linolelaidate	49.57	61.35	47.23	57.17	48.65	70.26	74.86	80.09	44.36
12.46	C18:3 n-6	methyl* cis*,*cis*,*cis*-6,9,12-octadecadienoate	methyl γ-linolenate	0.05	-	14.63	0.41	12.26	-	0.40	-	-
12.90	C18:3 n-3	methyl* cis*,*cis*,*cis*-9,12,15-octadecatrienoate	methyl α-linolenate	31.42	9.28	14.61	22.54	1.11	1.41	0.95	0.53	28.42
13.86	C20:0	methyl eicosanoate	methyl arachidate	0.77	0.62	0.12	-	-	-	0.50	-	< 0.01
14.19	C20:1	*methyl cis*-11-eicosenoicoate	methyl gondoate	0.33	0.21	2.46	-	-	-	-	-	<0.01
14.94	C20:2 n-6	methyl* cis*,*cis*-11,14-eicosadienoate	none	-	-	-	-	-	-	0.62	-	-

**Table S2:** Average mass fractions of sugars present in berry seed samples and their retention times (*t*_R_)

*t*_R_/min	Sugars and poliols	*w*(sugar)/(mg/kg)								
Strawberry	Blackberry	Black currant	Raspberry	Gooseberry	Chokeberry	Goji berry	Cape gooseberry	Blueberry
2.21	Erythritol	2.93	1.50	4.19	0.98	2.54	1.71	3.76	2.48	1.36
2.36	Glycerol	12.45	17.45	26.21	11.80	14.72	16.53	29.48	12.78	9.03
2.60	Arabinitol	0.58	3.93	4.23	0.87	0.99	1.30	0.90	0.63	0.65
2.72	Sorbitol	0.95	3.22	1.42	2.01	1.46	9.01	1.69	0.82	2.53
3.05	Galactitol	0.54	1.00	1.17	0.45	0.13	0.45	0.24	0.29	0.52
3.38	Mannitol	0.53	3.47	1.48	1.75	0.87	0.39	0.82	0.48	0.39
3.68	Trehalose	3.58	18.12	13.15	0.24	2.53	16.02	2.16	2.18	6.85
4.21	Ramnose	0.25	0.52	0.60	0.27	0.57	0.71	0.11	0.36	0.22
5.04	Arabinose	2.26	4.47	2.41	1.80	3.46	7.13	3.92	1.86	2.21
5.36	Xylose	8.65	2.66	0.67	0.63	0.99	1.79	0.22	0.09	0.28
5.90	Glucose	75.58	148.60	61.67	88.02	61.99	133.66	76.88	67.69	70.21
6.88	Fructose	116.04	173.21	67.17	130.40	105.82	84.46	77.56	68.96	79.38
7.90	Melibiose	1.89	4.71	4.04	10.67	5.50	2.66	4.82	1.51	25.92
8.39	Isomaltose	5.47	0.45	1.13	1.30	0.67	0.14	0.15	0.93	0.74
9.70	Sucrose	126.84	111.42	129.76	144.45	155.79	97.80	162.16	121.23	91.17
10.72	Celibiose	1.63	0.62	3.26	0.86	2.26	0.75	1.29	2.16	0.84
13.74	Melesitose	4.09	0.87	3.52	0.39	1.04	1.84	3.74	7.77	1.72
14.01	Gentiobiose	2.89	0.41	3.04	0.20	0.49	0.69	2.45	2.62	1.34
14.35	Turanose	6.60	2.29	6.16	3.53	4.59	12.60	15.33	12.74	7.04
14.62	Raffinose	4.78	2.35	4.75	3.09	7.09	7.22	6.68	3.96	3.13
14.93	Isomaltotriose	0.82	0.73	1.91	0.32	1.43	0.60	0.73	0.76	0.54
15.82	Maltose	3.87	2.74	4.33	2.85	3.57	2.67	7.28	3.14	2.49
18.11	Panose	5.91	55.41	8.88	13.18	18.61	19.40	20.63	8.93	8.37
19.43	Maltotriose	0.41	0.44	1.87	0.18	0.49	0.53	0.21	0.31	0.47
24.38	Maltoteteraose	3.00	1.39	7.88	1.32	2.25	1.76	1.61	2.20	3.98
24.68	Maltopentaose	2.70	1.31	7.08	1.11	2.09	1.44	1.06	1.90	3.64
25.03	Maltohexaose	3.99	1.89	9.82	1.62	3.05	2.32	1.30	2.72	4.93
25.39	Maltoheptaose	4.42	2.16	10.71	1.78	2.88	2.35	1.12	3.10	5.43
